# Improvement of Suspected Paraneoplastic Large-Vessel Vasculitis after Breast Cancer Resection: A Case Report

**DOI:** 10.70352/scrj.cr.26-0408

**Published:** 2026-07-29

**Authors:** Yuki Asaka, Haruhito Kinoshita, Hanae Matsuda, Saeko Henmi, Yuko Kikukawa, Ayako Gose, Mai Nishimoto, Asuka Kochi, Chika Watanabe, Koji Takada, Yukie Tauchi, Kana Ogisawa, Tamami Morisaki, Kenichi Kohashi, Shinichiro Kashiwagi

**Affiliations:** 1 Department of Breast Surgical Oncology, Osaka Metropolitan University Graduate School of Medicine, Osaka, Osaka, Japan; 2 Department of Pathology, Osaka Metropolitan University Graduate School of Medicine, Osaka, Osaka, Japan

**Keywords:** breast cancer, paraneoplastic vasculitis, large-vessel vasculitis, aortitis, tumor resection, paraneoplastic syndrome

## Abstract

**INTRODUCTION:**

Paraneoplastic vasculitis is an uncommon manifestation of malignancy and has been reported mainly in association with lung cancer, renal cell carcinoma, and hematologic malignancies. Breast cancer-associated vasculitis is rare, and presentation as large-vessel vasculitis (LVV) is particularly uncommon. We report a rare case of suspected paraneoplastic LVV occurring concurrently with breast cancer, in which inflammatory activity stabilized after surgical resection of the primary tumor.

**CASE PRESENTATION:**

A 54-year-old woman presented with pain in the left upper limb and left thigh. CT suggested left breast cancer with ipsilateral axillary lymph node metastasis. PET/CT showed fluorodeoxyglucose uptake in the left breast lesion and left axillary lymph node, as well as in multiple large vessels, including the carotid arteries, subclavian arteries, aorta, iliac arteries, and femoral arteries. Laboratory testing revealed an elevated C-reactive protein level of 10.93 mg/dL, whereas immunoglobulin A and antineutrophil cytoplasmic antibodies were within the normal ranges. There was no evidence of infection, autoimmune disease, or drug-induced vasculitis. Because breast cancer-associated paraneoplastic LVV was suspected, prednisolone was initiated at 30 mg/day, resulting in rapid improvement in symptoms and inflammatory response. The patient subsequently underwent breast-conserving surgery with axillary lymph node dissection while receiving prednisolone at 15 mg/day. Pathological examination revealed invasive ductal carcinoma, pT1c pN1 cM0, stage IIA, luminal A subtype. After surgery, prednisolone was tapered to 5 mg/day without recurrence of vasculitis, and inflammatory activity remained controlled during follow-up.

**CONCLUSIONS:**

LVV occurring concurrently with newly diagnosed breast cancer may represent a paraneoplastic manifestation after exclusion of autoimmune, infectious, and drug-induced causes. This case suggests that tumor resection may contribute to stabilization of inflammatory activity by reducing tumor-related antigenic or cytokine-mediated stimulation. Surgical treatment may therefore have a potential role not only in local oncological control but also in the management of paraneoplastic immune dysregulation in selected patients with resectable breast cancer.

## Abbreviations

ANCA
: antineutrophil cytoplasmic antibody
CH50
: total hemolytic complement activity
CRP
: C-reactive protein
ER
: estrogen receptor
ESR
: erythrocyte sedimentation rate
FDG
: fluorodeoxyglucose
G-CSF
: granulocyte colony-stimulating factor
HER2
: human epidermal growth factor receptor 2
ICI
: immune checkpoint inhibitor
IgA
: immunoglobulin A
LVV
: large-vessel vasculitis
MPO-ANCA
: myeloperoxidase-antineutrophil cytoplasmic antibody
PgR
: progesterone receptor
PR3-ANCA
: proteinase 3-antineutrophil cytoplasmic antibody
RF
: rheumatoid factor
RS
: recurrence score
SS-A
: Sjögren’s syndrome-related antigen A

## INTRODUCTION

Paraneoplastic syndromes are systemic disorders caused by malignancy-related immune, hormonal, or cytokine-mediated mechanisms rather than by direct tumor invasion or metastasis.^1)^ Among them, paraneoplastic vasculitis is uncommon and has been described more frequently in association with hematologic malignancies, lung cancer, renal cell carcinoma, and other solid tumors.^2)^ The proposed mechanisms include immune-complex deposition, cross-reactive immune responses against tumor-associated antigens, and cytokine-mediated vascular inflammation.^1,2)^

Vasculitis associated with malignancy most commonly involves small vessels and often presents as cutaneous leukocytoclastic vasculitis or IgA vasculitis.^2–4)^ In contrast, LVV associated with breast cancer is extremely rare. In patients with breast cancer, LVV or aortitis has been reported mainly in relation to anticancer treatments, including G-CSF, taxane-containing chemotherapy, and ICIs.^5–7)^ Therefore, the occurrence of LVV at the time of breast cancer diagnosis, before exposure to systemic anticancer therapy, raises a challenging differential diagnosis that includes primary autoimmune vasculitis, infection, drug-induced vasculitis, and paraneoplastic vasculitis.

Here, we report a rare case of breast cancer accompanied by suspected paraneoplastic LVV involving the aorta and major branch arteries. The inflammatory activity stabilized after surgical resection of the primary breast tumor, allowing tapering of corticosteroid therapy without recurrence of vasculitis. This case highlights the importance of considering paraneoplastic vasculitis in the differential diagnosis of LVV associated with newly diagnosed breast cancer and suggests that tumor resection may contribute to the control of paraneoplastic immune dysregulation.

## CASE PRESENTATION

A 54-year-old woman with no relevant medical history presented to a local hospital with pain in the left upper limb and left thigh. Initial imaging studies suggested left breast cancer with ipsilateral axillary lymph node metastasis and possible aortitis, and she was referred to our institution for further evaluation and treatment. On physical examination, no specific skin lesions suggestive of cutaneous vasculitis were observed. Laboratory tests showed a marked inflammatory response, with a CRP level of 10.93 mg/dL and an ESR of >100 mm/h. Serum complement activity was mildly elevated, with a CH50 level of 60 U/mL. IgA was within the normal range at 263 mg/dL. RF, anti-SS-A antibody, MPO-ANCA, and PR3-ANCA were negative or within the normal ranges. There was no clinical evidence of infection, and the patient had not received G-CSF, chemotherapy, ICIs, or other drugs known to induce LVV.

**Fig. 1:**
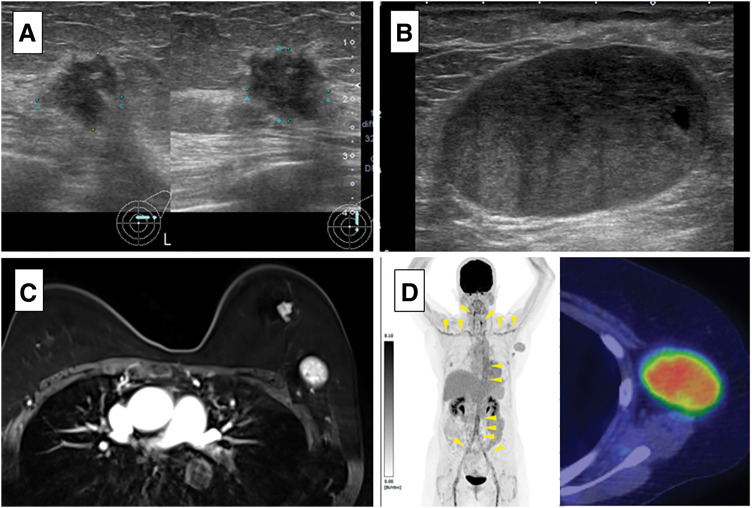
Imaging findings of breast cancer and LVV. (**A**) Breast ultrasonography showed an irregular hypoechoic mass in the upper outer quadrant of the left breast. (**B**) Ultrasonography showed an enlarged left axillary lymph node. (**C**) Contrast-enhanced MRI demonstrated an enhancing mass in the left breast and an enlarged ipsilateral axillary lymph node. (**D**) FDG PET/CT showed abnormal uptake in the left breast lesion, left axillary lymph node, and multiple large vessels, consistent with LVV (yellow arrowheads). SUVmax values were not available from the archived PET/CT report. FDG, fluorodeoxyglucose; LVV, large-vessel vasculitis

Breast ultrasonography revealed a 1.4-cm hypoechoic mass in the upper outer quadrant of the left breast and an enlarged left axillary lymph node measuring 3.8 cm. Contrast-enhanced MRI showed an enhancing mass measuring 1.1 cm in the left breast and a single enlarged axillary lymph node. PET-CT demonstrated FDG uptake in the left breast lesion and left axillary lymph node. In addition, abnormal uptake was observed along multiple large vessels, including the carotid arteries, subclavian arteries, aorta, and arteries extending from the common iliac to the femoral regions, consistent with LVV (**Fig. 1**). Quantitative SUVmax values were not available from the archived PET/CT report; therefore, the PET/CT findings were assessed qualitatively based on the distribution of FDG uptake and the corresponding clinical inflammatory activity.

Core needle biopsy of the breast tumor revealed invasive ductal carcinoma. Immunohistochemical analysis showed ER positivity, weak PgR positivity, HER2 negativity, and a low Ki-67 labeling index of 5%. The clinical diagnosis was left breast cancer, cT1N1M0, clinical stage IIA, luminal type, complicated by suspected paraneoplastic LVV.

The patient was admitted to the rheumatology department and treated with oral prednisolone at an initial dose of 30 mg/day. Her limb pain and inflammatory response improved promptly after corticosteroid therapy. Because the onset of vasculitis coincided with the diagnosis of breast cancer, autoimmune markers were negative, and no infectious or drug-induced cause was identified, breast cancer-associated paraneoplastic LVV was suspected. Takayasu arteritis and other primary autoimmune vasculitides were considered in the differential diagnosis; however, the patient had no previous history of chronic vascular symptoms, and serological findings did not suggest IgA vasculitis, ANCA-associated vasculitis, or connective tissue disease-associated vasculitis. After multidisciplinary discussion, upfront surgery was selected to remove the presumed antigenic source while controlling vascular inflammation with corticosteroid therapy.

**Fig. 2:**
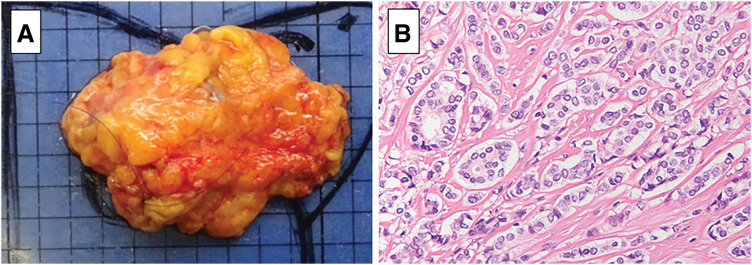
Surgical and pathological findings. (**A**) Gross appearance of the resected breast specimen after breast-conserving surgery. (**B**) Histopathological examination revealed invasive ductal carcinoma composed of atypical epithelial cells forming invasive nests within the fibrous stroma. Hematoxylin and eosin staining, original magnification ×200.

Approximately 2 months after the initiation of corticosteroid treatment, the patient underwent breast-conserving surgery with axillary lymph node dissection while receiving prednisolone at 15 mg/day. Perioperatively, the potential risks of infection, impaired wound healing, and flare of vascular inflammation were reviewed with the rheumatology team, and surgery was performed after clinical and laboratory inflammatory activity had improved under corticosteroid therapy. The postoperative course was uneventful. Pathological examination revealed invasive ductal carcinoma measuring 15 mm in diameter. One of 9 dissected axillary lymph nodes contained metastasis. The final pathological stage was pT1c pN1 cM0, stage IIA. Immunohistochemical findings were ER positive, PgR positive, HER2 score 1+, and Ki-67 labeling index 5%, consistent with the luminal A subtype (**Fig. 2**).

The RS determined by the 21-gene assay was 3. Postoperatively, the patient received adjuvant radiotherapy followed by endocrine therapy with anastrozole at 1 mg/day. Corticosteroid therapy was gradually tapered after surgery. At 6 months after surgery, the prednisolone dose had been reduced to 5 mg/day, and no recurrence of vasculitis or breast cancer was observed. The inflammatory activity remained clinically stable during follow-up. At the latest follow-up, the patient remained on prednisolone 5 mg/day, with no recurrence of limb pain and no rebound elevation of inflammatory markers. No additional immunosuppressive agent was required. Follow-up PET/CT specifically evaluating vasculitis was not performed because symptoms and inflammatory activity remained controlled; therefore, direct quantitative comparison of vascular FDG uptake before and after treatment was not available.

## DISCUSSION

We report a rare case of suspected paraneoplastic LVV occurring concurrently with newly diagnosed breast cancer and improving after surgical resection of the primary tumor. This case has 3 important clinical implications. First, LVV should be considered in patients with malignancy who present with unexplained systemic inflammation and vascular symptoms. Second, breast cancer-associated paraneoplastic vasculitis, although rare, may involve not only small vessels but also the aorta and its major branches. Third, surgical removal of the primary tumor may contribute to stabilization of paraneoplastic immune dysregulation when the malignancy is considered the potential trigger.

LVV is classically defined as vasculitis predominantly affecting the aorta and its major branches.^8)^ In the present case, FDG uptake was observed in multiple large vessels, including the carotid arteries, subclavian arteries, aorta, and arteries extending from the common iliac to the femoral regions. This distribution was compatible with LVV rather than localized vascular inflammation. ^18^F-FDG PET/CT is useful for evaluating the extent and activity of LVV, particularly when inflammatory activity is systemic or when multiple vascular territories are involved.^9,10)^ In our patient, the simultaneous visualization of the breast tumor, metastatic axillary lymph node, and multifocal vascular inflammation was helpful for recognizing the coexistence of malignancy and LVV.

The diagnosis of paraneoplastic vasculitis remains challenging because no disease-specific biomarker has been established. In general, a paraneoplastic relationship is supported by the close temporal association between vasculitis and malignancy, exclusion of other causes, and a parallel clinical course between tumor control and vasculitis activity.^2,11)^ In the present case, the onset of vascular symptoms and inflammatory activity coincided with the diagnosis of breast cancer. Autoimmune markers, including antineutrophil cytoplasmic antibodies, were negative or within the normal ranges, and there was no clinical evidence of infection. In addition, the patient had not received chemotherapy, G-CSF, ICIs, or other drugs known to induce LVV before the onset of symptoms. Takayasu arteritis was an important differential diagnosis because it can affect the aorta and its major branches. However, the patient was 54 years old, had no documented history of chronic or relapsing vascular symptoms before the breast cancer diagnosis, and had unremarkable serological findings, including MPO-ANCA, PR3-ANCA, RF, anti-SS-A antibody, and IgA. Furthermore, there were no clinical features suggesting IgA vasculitis, ANCA-associated vasculitis, connective tissue disease-associated vasculitis, infection, or drug-induced vasculitis. Although primary LVV cannot be completely excluded, these findings supported the possibility of breast cancer-associated paraneoplastic LVV.

Most malignancy-associated vasculitides reported in solid tumors involve small vessels, such as leukocytoclastic vasculitis or IgA vasculitis.^2–4,11)^ Breast cancer-associated paraneoplastic vasculitis is particularly uncommon, and reported cases have mainly included cutaneous small-vessel vasculitis or ANCA-associated vasculitis.^3,4)^ In contrast, LVV in patients with breast cancer has been described mainly as a treatment-related adverse event. G-CSF- or chemotherapy-associated aortitis has been increasingly recognized in breast cancer treatment, particularly in patients receiving pegfilgrastim-containing dose-dense regimens.^5–7,12,13)^ More recently, ICI-associated vasculitis, including LVV, has also been reported in patients with cancer.^14,15)^ The present case is distinct from these treatment-related forms because LVV was already present at the time of breast cancer diagnosis, before exposure to systemic anticancer therapy.

The most clinically relevant feature of this case was the stabilization of inflammatory activity after tumor resection. Corticosteroid therapy produced an initial improvement in symptoms and laboratory inflammation, allowing surgery to be performed safely. However, after breast-conserving surgery and axillary lymph node dissection, prednisolone could be tapered to a low dose without recurrence of vasculitis. Importantly, the improvement in vasculitis should not be attributed to tumor resection alone, because corticosteroid therapy was initiated before surgery and likely contributed substantially to the early improvement in symptoms and inflammatory markers. Although the contribution of corticosteroid therapy cannot be separated from that of surgery, the postoperative course suggests that removal of the tumor may have reduced the antigenic or cytokine-mediated stimulus driving vascular inflammation. This clinical course is consistent with the concept that treatment of the underlying malignancy is central to the management of paraneoplastic syndromes.^1,11)^

This case also has implications for surgical decision-making. In patients with breast cancer complicated by active vasculitis, clinicians may hesitate to proceed with surgery because of systemic inflammation and corticosteroid use. Perioperative management requires balancing the need for immunosuppression to control vascular inflammation against the potential risks of infection, delayed wound healing, and unnecessary postponement of oncological treatment. In the present case, surgery was performed after improvement of symptoms and inflammatory activity while the prednisolone dose had been reduced to 15 mg/day, which allowed safe tumor resection without postoperative complications. However, when infection, primary autoimmune vasculitis, and drug-induced vasculitis have been carefully excluded and the primary tumor is considered a potential trigger, timely surgical resection may serve not only as oncological local control but also as a form of immunological source control. Multidisciplinary collaboration among breast surgeons, rheumatologists, radiologists, and pathologists is essential to determine the timing of surgery and the perioperative corticosteroid strategy.

This report has several limitations. First, this is a single case, and a causal relationship between breast cancer and LVV cannot be proven. Second, vascular tissue was not histologically examined; therefore, the diagnosis of vasculitis was based on clinical, laboratory, and imaging findings. Third, corticosteroid therapy had been initiated before surgery, which may have contributed substantially to the improvement in inflammatory activity; therefore, the apparent postoperative stabilization should be interpreted cautiously and cannot establish an independent therapeutic effect of tumor resection. Fourth, quantitative SUVmax values on baseline PET/CT were not available, and follow-up PET/CT was not performed specifically to reassess vascular inflammation; consequently, imaging-based changes in vasculitis activity could not be objectively compared. Finally, the follow-up period remains limited, and longer observation is required to confirm the absence of vasculitis recurrence and breast cancer relapse. Nevertheless, the close temporal association, exclusion of other major causes, and stabilization after tumor resection support the interpretation that this case represents suspected paraneoplastic LVV associated with breast cancer.

## CONCLUSIONS

Breast cancer-associated paraneoplastic LVV is extremely rare but should be considered when LVV develops concurrently with newly diagnosed breast cancer, particularly before exposure to chemotherapy, G-CSF, or ICIs. In the present case, tumor resection was followed by stabilization of inflammatory activity and successful tapering of corticosteroid therapy without recurrence of vasculitis. This case suggests that surgical resection of the primary tumor may contribute to the control of paraneoplastic immune dysregulation in selected patients with resectable breast cancer.

## DECLARATIONS

### Availability of data and materials

All data generated or analyzed during this study are included in this published article.
